# Prevalence, Contamination Level, and Associated Factors of Methicillin-Resistant *Staphylococcus aureus* in Raw Cow Milk at Selected Districts of Gamo Zone, Southern Ethiopia

**DOI:** 10.1155/2023/6238754

**Published:** 2023-04-15

**Authors:** Edget Abayneh Alembo, Tomas Tonjo Torka

**Affiliations:** ^1^College of Agricultural Science, Animal and Health Science Department, Arba Minch University, Arba Minch, Ethiopia; ^2^Dita District Livestock and Fishery Department, Arba Minch University, Arba Minch, Ethiopia

## Abstract

*Staphylococcus aureus* is pathogenic bacterium contaminating milk and milk products causing bacterial food poisoning. In the current study sites, there is no information on methicillin-resistant *Staphylococcus aureus*. Thus, the current study sought to assess the risk factors that contribute to the contamination of raw cow milk, the bacterial load, and the prevalence of methicillin-resistant *Staphylococcus aurous*. A cross-sectional study was conducted, January to December, 2021, on randomly selected 140 milk samples from selling point of Arba Minch Zuria and Chencha districts. Fresh milk samples were processed and tested for bacterial load, bacterial isolation, and methicillin susceptibility patterns. Questionnaire survey was conducted on 140 producers and collectors to assess hygienic factors attributed to contamination of raw cow milk with *Staphylococcus aureus*. The overall prevalence of *S. aureus* was 42.1% (59/140) (95% confidence interval (CI): 34.80–51.40%). About 15.6% (22/140) of the milk samples assessed had the viable count and total *S. aureus* count higher than 5log cfu/mL with 5.3 + 1.68 and 1.36 + 1.7log cfu/ml^−1^ bacterial loads, respectively. The rate of isolation of *S. aureus* was significantly high in milk from highland than lowland (*p*=0.030). The multivariable logistic regression revealed that educational status (OR: 6.00; 95% CI: 4.01–8.07), picking one's nose while working on milk (OR: 1.41; 95% CI: 0.54–2.25), cleaning the milk can (OR: 4.5; 95% CI: 2.61–5.17), hand washing activities (OR: 3.4; 95% CI: 1.670–6.987), check for abnormal milk (OR: 2; 95% CI: 1.55–2.75), and container for milk (OR: 3; 95% CI: 0.12–0.67) were risk factors significantly associated with the occurrence of *S. aureus* in milk. In conclusion, the highest rate of resistance was observed to ampicillin (84.7%) and cefoxitin (76.3%). All isolates are resistant to at least two types of antimicrobial drugs, while 65.0% of the isolates were found to be multidrug-resistant. The higher prevalence, high load, and antimicrobial resistance of *S. aureus* indicate the higher public health risk due to the widespread consumption of raw milk in the area. Furthermore, consumers in the study area should be aware of the risks associated with consuming raw milk.

## 1. Introduction

The health effects of food-borne diseases are getting worse and worse in the twenty-first century [1]. Despite the fact that the issue is global, the poor world bears the heaviest weight. According to the estimations of the WHO, up to 30% of the population in the developed countries experiences food-borne illnesses each year, compared to up to 2 million deaths in the developing countries, with Ethiopia ranking second after Nigeria in terms of the health burden of these illnesses in African nations. This poses a serious threat to the health of the local population and has significant economic repercussions [2].

Foods of animal origin are linked to the bulk of food-associated outbreaks, accounting for 85% of them, with dairy accounting for 20% [3, 4]. The majority of milk vendors in Ethiopia, where the dairy industry is transitioning to a market-oriented system, purchases unpasteurized milk from various dairy farms to sell to customers [5]. In addition, a survey carried out in central Ethiopia revealed that 31.8% of the people of all ages consumed raw milk [6]. Since milk is regarded as a full meal for humans and serves as a medium for microbial growth, it is imperative that the products' hygiene standards are met [7].

In instance, bacterial infections and/or toxic compounds produced by various organisms growing in milk are the most frequent sources of safety concerns for milk consumers [8]. Milk from many sources may become contaminated with unwanted bacteria through improper handling and unclean processing procedures by milk handlers, making the milk unfit for direct consumption [9].

In both humans and animals*, Staphylococcus aureus* (*S. aureus*) is a commensal and opportunistic pathogen that can cause a wide range of illnesses [10]. One of the most prevalent food-borne infections at the moment is staphylococcal food poisoning, which is a serious issue for public health initiatives everywhere [11].

Humans can contract *S. aureus* from tainted cow's milk since it serves as the best growth medium for the bacteria [12]. The most concerning aspect of the situation is that tainted milk may include antimicrobial-resistant *S. aureus*, posing major health risks to consumers, and being acknowledged by international health organizations as one of the most important health concerns of the twenty-first century [13]. Methicillin-resistant *S. aureus* (MRSA) and other multidrug-resistant bacteria have become a serious public health concern, particularly in the developing nations, where there are few effective treatments for these strains [14].

Nearly all of the penicillin group's antibiotics, which are very thorough and still frequently used in both human and veterinary medicine, are ineffective against MRSA [15]. Overall, the situation is particularly concerning in underdeveloped nations like Ethiopia, where there is a high prevalence of infectious illnesses, a dearth of surveillance networks, lab capacity difficulties, and poor diagnostic practices [16]. The use of raw milk is customary in our study area's setting, particularly in the towns of Arba Minch and Chencha, and consumer demand is rising as a result of population expansion, rising per capita income, and urbanization. Numerous milk selling stations are opening up in the area daily to meet these demands. Therefore, it is crucial to have bacteriological quality, and *S. aureus* prevalence, antibiotic sensitivity patterns, and associated sources of contamination are all unknown, and there have been no prior studies in the area. This study therefore sought to evaluate the microbiological load of milk, *S. aureus* isolation, patterns of antimicrobial susceptibility, prevalence of MRSA, and related risk factors of contamination in Arba Minch and Chencha town of Gamo zone, southern Ethiopia.

## 2. Methods

### 2.1. Study Area

**Figure 1:**
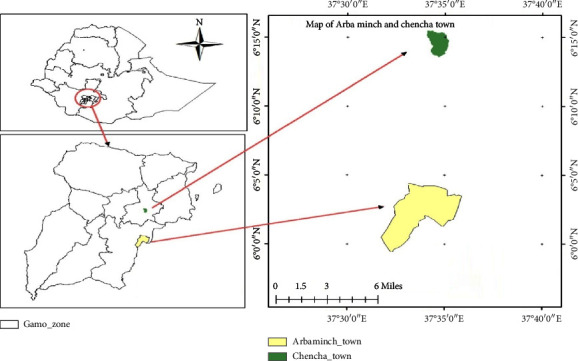
Map of study area (Gamo zone environment and forest protection biro).

Over the course of a year, a study was undertaken in the southern Ethiopian towns of Arba Minch and Chencha (January–December, 2021) (Figure 1). Arba Minch Town is the capital of the Gamo zone, located 276 kilometers from Hawassa, the SNNPR's capital, and 500 kilometers south of Addis Ababa. Chencha Town is one of the administrative towns in the Gamo zone, and it is situated 37 kilometers to the north of Arba Minch Town. They are lowlands with altitude of 500–1,000 m and highlands from 2,300–3,200 m above sea level, respectively, in terms of agroecology. Both settlements, particularly Chencha, are well renowned for having a strong potential for dairy cow production and for delivering milk to the towns of Arba Minch and other nearby communities. In both situations, it is customary for the locals to consume the raw milk and distribute any leftovers to nearby areas [17].

### 2.2. Study Population

Based on the milk owners' wishes and availability of producers and collectors, this study was conducted on 140 milk producers and collectors from 200 milk producers and collectors present at various selling points in the study district.

### 2.3. Study Design and Sampling Technique

In Gamo zone, southern Ethiopia, a cross-sectional study design was used to investigate the bacteriological quality of raw cow milk, estimate the prevalence of methicillin-resistant *S. aureus*, and assess associated risk factors of milk contamination. The study area was divided into two sections based on agroecological location: highland and lowland. Then, based on the information provided by the trade offices in both towns from 200 milk producers and collectors, a total of 140 milk samples were allocated proportionally to the study areas. Before collecting the milk samples and questionnaires were completed, and the milk samples, milk producers, and collectors were selected using a simple random sampling technique.

### 2.4. Sample Size Determination

The milk sample size was calculated using the single population proportion formula and the assumptions listed in the following [18]. *N* = *Z*^2^*P*_exp_ (1 − *P*_exp_)/*D*^2^, where *Z* = 1.96, *N* = sample size, *P*_exp_ = expected prevalence, and *D* = absolute precision. The previous study [19, 20] used a pooled isolation rate of 3.175% for methicillin-resistant *S. aureus*, with a 95% confidence level and a 5% margin of error. As a result, the minimum sample size (*n*) was determined to be 47, and the sample size was tripled to 140 to increase precision [21].

### 2.5. Sample Collection Methods

#### 2.5.1. Check List/Questionnaire

Before collecting milk samples, milk handlers were observed for basic hygiene practices such as the use of a hair cap, clean overcoat, and nose touching habit using a prepared checklist. A structured questionnaire was used to collect information on educational status, training attended, medical checkup, hand washing practices, milk container washing practices, and milk container type used.

#### 2.5.2. Milk Sample

The milk samples were drawn and handled with the utmost care to prevent accidental contamination. The samples were procured in sterile test tubes and delivered right away in an icebox to the Arba Minch University's Microbiology lab for the bacteriological analysis. Within 2–4 hours, the samples were evaluated [22].

### 2.6. Bacterial Load Analysis

Using a homogenizer, a 25 ml milk sample was homogenized in 225 ml buffered peptone water. The final homogenate resulted in a 1 : 10 dilution. Total viable count (TVC) and total *S. aureus* count (TSC) were evaluated using serial dilution up to 10^−6^ [23].

#### 2.6.1. TVC

0.1 ml of each serial dilution was spread onto plate count agar (PCA) (Himedia) and incubated for 24 hours at 37°C. Following incubation, distinct colonies ranging from 30 to 300 on PCA were counted and calculated using the following formula [24], which was then expressed in colony forming units per milliliter (cfu/ml). Finally, it was classified using criteria established by the hazard analysis and risk assessment in the management of food safety and quality for bacteriological limit standards for human consumption [25].

(1) $$\begin{array}{c} \frac{CFU}{ml}=\frac{\sum C}{V\times 1.1\times d}\text{,} \end{array}$$

where ∑*C* is the sum of the colonies counted from two successive dilutions on the two Petri dishes; *V* is the volume of inoculum placed in each Petri dish, which was 0.1 (in milliliters); and *d* is the first dilution retained.

#### 2.6.2. TSC

On mannitol salt agar, 0.1 ml of each successive dilution was applied. Isolated separate colonies from 30 to300 were enumerated and estimated using the previously indicated technique after 24 hours of incubation at 37°C. The outcomes were then categorized according to the criteria established by risk assessment and hazard analysis in the management of food safety and quality for human consumption [25].

### 2.7. Isolation of Bacteria

Based on colony features of the organisms, such as growth on mannitol salt agar and Gram's stain, *S. aureus* was initially identified. The next step was to validate *S. aureus* using biochemical tests such as coagulase test, catalase test, indole production, methyl red test, Voges–Proskauer reaction, urease production, citrate utilization, and sugar fermentation [26].

### 2.8. Testing for Antimicrobial Susceptibility

According to Clinical Laboratory Standards Institute (CLSI) recommendations, an antibacterial susceptibility test was conducted using the Kirby Bauer disc diffusion method on Muller–Hinton agar (Oxoid, Basingstoke, England). To achieve turbidity equivalent to 0.5 MacFarland standards per CLSI, three to five morphologically similar and fresh bacterial colonies were suspended on distilled sterile water suspension. On Mueller–Hinton agar, inoculum was equally dispersed across the surface using the sterile cotton swab as the seeding tool. After 15 minutes of inoculation, the antibiotic discs were placed to the medium's surface. Plates were then incubated at 35°C for 24 hours. The diameters of the inhibition zones surrounding the discs were then measured using a ruler and in accordance with the standards table outlined in Clinical and Laboratory Standards Institute to the nearest millimeter [27].

### 2.9. Detection of MRSA

According to CLSI [27], cefoxitin resistance was used to phenotypically identify MRSA. It is obvious that the isolated *S. aureus* organism was transferred to Mueller–Hinton agar (Oxoid, England) by making a suspension turbidity that was equal to 0.5 McFarland standards. The antibiotic cefoxitin disc was then positioned on the media. Methicillin-sensitive and methicillin-resistant *S. aureus* were identified based on the interpretation of the zone of inhibition during a 24-hour incubation period.

### 2.10. Data Quality Assurance

By adhering to the established standard operating procedure (SOP), data quality was guaranteed from data collection through final laboratory identification (SOP). The efficiency of the generated media was assessed by injecting control strains of *S. aureus* ATCC 29213 obtained from the Ethiopian Public Health Institute (EPHI), the effectiveness of the produced media was evaluated (EPHI). The manufacturer's instructions were followed for preparing the culture media, and the sterility was confirmed by incubating 5% of the finished product at 37°C for 24 hours while monitoring bacterial growth. Batches of the media that exhibit the growth were thrown out and remade.

### 2.11. Data Analysis

Using SPSS version 21 software, data were gathered, inputted, cleaned up, and analyzed in accordance with the study's goals. At a 95% confidence interval and 5% margin of error, estimation of proportions was performed to summarize the prevalence and antibiotic susceptibility patterns of *S. aureus* and MRSA. Text and tables were used to give the descriptive summary. In order to get odds ratios and confidence intervals for statistically related variables, the multivariate logistic regression analysis was used.

## 3. Results

### 3.1. Milk Handlers' Sociodemographic Characteristics and Hygienic Practices at Milk Selling Points

This study had 140 participants and a 100% response rate. More than 50% of the workers had completed elementary school, and the majority of the participants (65.7%) were females. It was discovered that 42.9% of participants pick their noses while working; 85% and 80%, respectively, did not wear a clean hair cup and wore a gown (Table 1).

### 3.2. Aerobic Bacterial Load Assessment

According to the current study, all 140 milk samples tested for bacterial load were positive for aerobic mesophilic bacteria, and 22 (15.6%) of those samples had bacterial loads that were too high in terms of TVC for human consumption. It also revealed that the log TVC's overall mean value was 5.3, with a standard deviation of 1.68. The current results' mean TVC did not meet the 5log cfu/mL requirement for raw milk intended for direct human consumption [28].

In terms of TSC, the mean value was 1.36 + 1.7log cfu/ml, with a contamination rate of 42.1%. According to the hazard analysis and risk assessment criteria for bacteriological limit standard for human consumption standards [25], it was extrapolated that 16 (11.4), 94 (67.2), 14 (10), and 16 (11.4) were found to be satisfactory, marginal, unsatisfactory, and potentially toxic for human consumption at a raw state (Table 2).

### 3.3. Isolation of *S. aureus*

Using colony morphology on culture, gram stain, and biochemical characteristics, the percentage of *S. aureus* isolates recovered from milk samples was calculated (Table 3). As a result, *S. aureus* was isolated in 42.1% (59/140) (95% of CI: 34.80–51.40%) cases. *S. aureus* is one of the most economically important food-borne pathogens found worldwide, and its presence in the result is a positive finding because some strains of *S. aureus* are capable of producing heat stable enterotoxins that are harmful to human health [2]. It was the most frequently isolated bacteria in the current study, with an isolation rate of 42.1%, i.e., 50% and 36.25% from highland and lowland milk samples, respectively. There was a significant difference in the isolation rate of *S. aureus* from lowland and highland with a *p* value less than 0.05.

### 3.4. Factors Contributing to Milk's Bacterial Contamination at the Point of Sale

After examining all of the study variables using a univariable logistic regression, the variables with a *p* value of less than 0.25 were used in the current study's multivariable logistic regression analysis. The current study's multivariate logistic regression discovered that *S. aureus* recovery was significantly correlated with milk handlers' educational level, hand washing practices, milk container cleaning activities, milk container type, and physical abnormal milk checking status, with *p* values 0.05. However, the variables sex of respondent, wearing hair cup, wearing gown, health check per year, and hygienic food handling training were not statistically associated at 95% confidence interval (Table 4).

### 3.5. Isolate Antibiogram Susceptibility Profiles

Antibacterial susceptibility patterns observed in the current study revealed that significant antibacterial resistance was detected among *S. aureus* isolates from milk, with a high rate of resistance to penicillin (84.7%), cefoxitin (76.3%), and gentamcin (50.9%) (Table 5). In terms of MRSA, 76.3% of *S. aureus* isolates demonstrated a zone of inhibition of 21 mm on the cefoxitin disc diffusion assay (Table 5) and were phenotypically extrapolated as methicillin-resistant *S. aureus*.

According to CLSI [27], the microorganisms in the current investigation were classified as MDR if they were resistant to three or more antibiotic classes. 83% of *S. aureus* isolates were discovered to be MDR (Table 6).

## 4. Discussion

More than half of the workers in the current study (65.7 percent) were female, and the majority had finished elementary school. In contrast, a study in a different region of Ethiopia found that milk handlers were mostly male [5]. According to the current findings, approximately 43% of the participants were only using water to wash their hands and milk containers. This is consistent with previous research conducted in Tigray, northern Ethiopia [5].

According to Bereda et al. [7], several other studies among food handlers in various areas of Ethiopia found that food handlers were vehicles for disease-causing microorganisms. Differences in food hygiene practice level could be attributed to differences in the study tool used, the time of study, and variations in sociodemographic and socioeconomic status. Poor hygiene may have contributed to microbial contamination of milk, and a lack of ongoing training and a low education level may have contributed to a lack of food hygiene knowledge regarding a number of critical aspects of safe food production [29].

The current study's mean TVC did not meet the 5log cfu/mL requirement for raw milk intended for direct human consumption [28] Similarly, higher mean TVC values (6.36–9.82 log cfu/mL) were discovered in various Ethiopian regions [7, 30]. The increased count in milk could be attributed to poor hygiene practices used during manufacturing and subsequent handling [31].

The current study found that 10.7% of total samples exceeded the recommended level of TSC load limit, with 11.4% detected in toxic range, indicating a risk to consumer health when consumed in raw form. The mean count was found to be within the range of the recommended level of TSC load for human consumption, and it agreed with a previous study conducted in Iran [32]. *S. aureus* in milk can come from both animal and human sources [33].

Previous research in Nepal found that 25%, 30%, and 45% of milk samples were satisfactory, fairly satisfactory, or poor quality for human consumption [8]. In general, the high bacteria counts observed in the current study were attributed to a lack of milk hygiene awareness, a low level of educational status, a lack of training on clean milk production, poor transportation conditions, poor hygiene of milking utensils, poor sanitation, milker's hands, and lack of hygiene in and around milking environments, as well as milk processing and handling [34].

The current study discovered an overall 42.1% prevalence of *S. aureus* in milk, which was consistent with previous studies in Bishoftu, Ethiopia [32, 35], but higher than the corresponding values published in other studies in central Oromia (16%) [36, 37], Ethiopia. The differences in isolation rates could be attributed to changes in handling practices, posthandling of the milk, and general hygiene standards maintained at various stages of the milk processing chain. Furthermore, variations in the isolation rate of bacterial isolates could be attributed to sample size, study design, methodology used, and geographical location [28]. Improving food handler and equipment hygiene, as well as the use of cold chain facilities, was required in the milk chain to protect consumers from milk-borne hazards, and controlling *S. aureus* in dairy products is required for commercial and profitable small-scale cow farming to improve milk quality for consumers and dairy industries.

Frequent use of milk product containers without adequate cleaning may increase *S. aureus* contamination of the product, and the use of plastic and traditional containers (clay pots) can be a potential source of milk contamination because they allow bacteria to multiply on milk contact surfaces between milking processes. Due to heat, their ability to form biofilm in collecting and storage tanks, and their resistance to insufficient cleaning, *S. aureus* persists and multiplies in milk buckets [38]. The most likely cause of the high prevalence of *S. aureus* is a lack of routine food-borne pathogen prevention and control practices implemented by farms, milk collection centers, and milk product handlers [39].

In terms of *S. aureus* contamination, it was discovered that washing milk containers with only water increased the likelihood of milk contamination at selling points by 4.5 times compared to using both detergent and water (OR = 4.5; 95% CI = 2.61–5.17) and by 3.4 times compared to washing hands with both detergent and water (OR = 3.4; 95% CI = 1.67–6.98). This finding was consistent with previous research from central Ethiopia, which found that washing milk containers with water alone increased the likelihood of milk contamination [40]. Our research also revealed the possibility of *S. aureus* contamination of milk at facilities with trained staff in sanitary food handling [27].

According to a data abstract from the Federal Democratic Republic of Ethiopia's Central Statistical Authority, the danger is decreased by 40% when compared to untrained milk workers. This conclusion was supported by a previous study from Addis Ababa, Ethiopia, which indicated that workers with greater levels of education had a 3.5 times higher likelihood of eliminating bacterial contamination in milk [41]. The current study found that milk contamination with *S. aureus* is three times more likely in synthetic jerkan than in aluminum-coated containers (OR = 3; 95% CI = 1.25–6.72). Donkor et al. [42] discovered that the use of plastic milk containers is a potential hygienic factor associated with bacterial contamination of milk because it provides suitable environments for biofilm forming bacteria.

According to the current findings, the odds of milk contamination with *S. aureus* are two times higher in selling points that did not check abnormal milk (OR = 2; 95% CI = 1.55–2.75). A similar study found that the hygiene of milk product containers is significantly associated with the occurrence of *S. aureus* due to the use of low-quality milk product containers. The prolonged use of low-quality plastic materials for handling milk products was common in milk collection centers and among farmers presenting milk products to the selling point. The safety of milk and milk products is determined by the utensils used for milking and storage [36].

This could be explained by the heat-induced proliferation of *S. aureus*, their ability to form biofilm in milk product containers, and their resistance to inadequate cleaning [39]. Milk and milk products can be contaminated after heat treatment due to poor hygiene of milk product containers, and the main sources of contamination are infected food handlers, as well as infections of animal origin [43]. The quality of milk and milk products is determined by the equipment used for milking, collecting, and storing [44].

In the current study, *S. aureus* showed high resistance to penicillin (84.7%), cefoxitin (76.3%), and gentamicin (50.9%). The current study agreed with previous studies in Ethiopia that found a high rate of penicillin resistance [33, 45]. The current study also showed 30.5% of *S. aureus* isolates were resistance to tetracycline which was similar to report of 32.5% in Holeta, Central Ethiopia [39]. This was lower than the previously reported high resistance (40%–82.2%) [46–48]. The current findings show that *S. aureus* was exposed to antibiotics and that the observed patterns reflect their use in the study area. Another possible explanation for the observed pattern is the availability and cost of these medications. It was discovered that these medications are generally available from agrovet wholesalers as well as human pharmacies and can be obtained without a prescription from an authorized institution [49, 50].

According to the findings of this study, 83% of *S. aureus* tested were multidrug resistant (MDR). The antimicrobial susceptibility tests revealed that the isolates exhibited general multidrug resistance characteristics (penicillin, cefoxitin, gentamicin, and tetracycline). This is consistent with the findings of [51] other studies that found a higher prevalence of multidrug-resistant *S. aureus* (60–70%) in raw milk from dairy cows in India. However, the current study discovered significantly more resistance than the previous Indian study (1.7%) [19].

This variation could be attributed to the frequent use of different lactams in the study areas, which may have contributed to the selection of resistant strains [49]. The emergence of drug resistance poses a public health risk because food-borne outbreaks may be difficult to treat, and the group of MDR *S. aureus* in the food supply serves as a reservoir for communicable resistant genes [52]. This could be attributed to the erratic and extensive use of antibacterial drugs without prior antimicrobial susceptibility testing. Such antimicrobial-resistant organisms can endanger both animals and humans' health. Antimicrobial resistance in *S. aureus* from bovine mastitis is currently increasing [38, 51].

## 5. Conclusion and Recommendations

The results of the current investigation showed that the raw milks given in the study area had higher bacterial loads than suggested standards, which is an indication of ineffective milk management procedures. In addition, it discovered 42.1% *S. aureus* with a high percentage of MDR (83%) that poses significant risks to the public's health. Our finding's 76.3% MRSA identification raises the possibility of resistant germs spreading to people and the environment. According to our research, *S. aureus* contamination of milk at the selling point was also related to the educational level of milk handlers, hand washing practices, milk container cleaning practices, milk container type, and physical abnormal milk checking status. To better comprehend and prevent the establishment of resistant bacterial strains linked to milk, the authors recommended that multidisciplinary surveillance programs be put into place. Programmed monitoring and inspection of milk for proper hygiene, handling, and sanitary practices by professionally qualified food safety officers should be warranted.

## Figures and Tables

**Table 1:** Hygiene behaviors and sociodemographic traits.

Variables	Categories	Frequency (*n*)	Percentage (%)
Sex	Male Female	48 92	34.3 65.7

Level of education	Illiterate 1–8 9–12 College and above	20 76 24 20	14.3 54.3 17.1 14.3

Attended training on food hygiene	Yes No	12 128	8.6 91.4

Putting on a hair cap	Yes No	20 120	14.3 85.7

Putting on a robe or apron	Yes No	28 112	20 80

Annual health examination	Yes No	8 132	5.7 94.3

Picking one's nose while handling milk	Yes No	60 80	42.9 57.1

Cleaning the milk can	Water and detergent Water only	81 59	57.9 42.1

Hand cleaning	Water and detergent Water only	77 63	55 45

Check for abnormal milk	Yes No	88 52	62.9 37.1

Container for milk	Vinyl jerkan Milk tank with an aluminum coating	96 44	68.6 31.4

**Table 2:** Bacteriological contamination and bacterial load of raw cow milk collected in Arba Minch and Chencha town, Gamo zone.

Geographical location sample sources	Total tested samples	*Growth status*	*Bacterial load (log10 cfu/g)*			*Acceptability of the milk under consideration based on microbiological load*			
		Positive sample nos. (%)	Min	Max	Mean ± SD cfu/g	Satisfactory no. (%)	Marginal no. (%)	Unsatisfactory no. (%)	Potential risk no. (%)
TVC	140	140	1.09	8.52	5.3 ± 1.68	54 (38.6)	64 (45.7)	22 (15.7)	
Highland	60	60 (100)	2.23	8.01	5.5 ± 1.37	21 (35)	30 (50)	9 (15)	N/A
Lowland	80	80 (100)	1.09	8.52	5.15 ± 1.8	33 (41.25)	34 (42.5)	13 (16.25)	N/A
TSC	140	59 (42.1)	0.00	4.99	1.36 ± 1.7	81 (57.9)	28 (20)	15 (10.7)	16 (11.4)
Highland	60	30 (50)	0.00	4.77	1.56 ± 1.7	30 (50)	15 (25)	10 (16.7)	5 (8.3)
Lowland	80	29 (36.25)	0.00	4.99	1.2 ± 1.7	51 (63.75)	13 (16.25)	5 (6.25)	11 (13.75)
Overall extrapolation						16 (11.4)	94 (67.2)	14 (10)	16 (11.4)

**Table 3:** Prevalence of bacterial isolates in the study area's raw cow milk samples.

Geographical locations	Number of samples	*S. aureus n* (%)	*X* ^2^	*p* value
Highland	60	30 (50)	0.906	0.03
Lowland	80	29 (36.25)		
Total	140	59 (42.1)		

**Table 4:** Multivariable logistic regression analysis of *S. aureus* contamination in milk.

Variables		*OR*	*CI at 95%*	*p value*
		*S. aureus*	*S. aureus*	*S. aureus*
Respondents' gender	Male	1.030	0.75–2.09	0.934
	Female	Ref	Ref	Ref

Educational attainment	Illiterate	6	4.01–8.07	0.000
	1–8	5	4.00–6.40	0.005
	9–12	2	1.04–3.89	0.6
	College and above	Ref	Ref	Ref

Participated in food hygiene training	Yes	0.6	0.14–1.69	0.37
	No	Ref	Ref	Ref

Putting on a hair cap	No	1.4	0.52–3.81	0.187
	Yes	Ref	Ref	Ref

Putting on a gown/apron	No	1.3	1.12–1.84	0.6
	Yes	Ref	Ref	Ref

Annual health examination	Yes	0.4	0.09–1.81	0.242
	No	Ref	Ref	Ref

Picking one's nose while working on milk	Yes	1.41	0.54–2.25	0.000
	No	Ref	Ref	Ref

Cleaning the milk can	Water only	4.5	2.61–5.17	0.000
	Water and detergent	Ref	Ref	Ref

Hand cleaning	Water only	3.4	1.67–6.98	0.001
	Water and detergent	Ref	Ref	Ref

Check for abnormal milk	No	2	1.55–2.75	0.000
	Yes	Ref	Ref	Ref

Container for milk	Vinyl jerkan	3	1.25–6.72	0.04
	Milk tank with an aluminum coating	Ref	Ref	Ref

Ref: reference point.

**Table 5:** Patterns of antibacterial susceptibility of bacterial isolates from raw cow milk in the study area.

Antibiotics	*S. aureus*		
	*S*	*I*	*R*
Penicillin	9 (15.3)		50 (84.7)
Cefoxitin	14 (23.7)		45 (76.3)
Gentamicin	29 (49.1)	0	30 (50.9)
Erythromycin	29 (49)	8 (13.5)	22 (37.3)
Tetracycline	27 (45.8)	14 (23.7)	18 (30.5)
Ciprofloxacin	57 (96.6)		2 (3.4)
Sulfamethoxazole	56 (94.9)	1 (1.7)	2 (3.4)
Clindamycin	56 (94.9)	0	3 (5.1)
Chloramphenicol	53 (89.8)	1 (1.7)	5 (8.5)

**Table 6:** Patterns of MDR of *S. aureus* isolates from raw cow milk in the study area.

MDR frequency	Antimicrobial's resistant pattern	No. of resistance isolates	Present (%)
Three total	PEN, CXT, GEN PEN, CXT, TET PEN, CXT, ERT PEN, GEN, CHL PEN, GEN, TET	9 3 8 2 3 25	18.3 6.2 16.3 4 6.2 51

Four total	PEN, CXT, GEN, TET PEN,CXT, GEN, SXTPEN, GEN, ERY, TET PEN, GEN, TET, CHL PEN, CXT, GEN, ERY PEN, CXT, TET, CIP PEN, CXT, ERY, TET	4 3 1 1 3 1 2 15	8.2 6.1 2 2 6.1 2 4.2 30.6

Five and above total overall	PEN, CXT, GEN, ERY, TET PEN, CXT, GEN, TET, CHL PEN, CXT, ERY, TET, CLI PEN, CXT, GEN, ERY, CLI	6 1 1 1 9 49	12.24 2.04 2.04 2.04 18.36 83

PEN, penicillin; CXT, cefoxitin; GEN, gentamicin; TET, tetracycline; ERY, erythromycin; CHL, chloramphenicol; CLI, clindamycin; CIP, ciprofloxacin; SXT, sulfamethoxazole.

## Data Availability

Online repositories contain the datasets used in this investigation. Information about the repository and repository names as well as the accession numbers at Zenodo is available at https://doi.org/10.5281/zenodo.7041886.

## Abbreviations:

MRSA: Methicillin-resistant *Staphylococcus aureus*
TVC: Total viable count
TSC: Total *Staphylococcus* count
MDR: Multidrug resistance
SNNPR: Southern Nations, Nationalities, and Peoples' Region
*S. aureus*: *Staphylococcus aureus*.

## Supplementary Materials

Supplementary Materials Table data 1. The entire necessary datum are included in the supplementary materials.
