# Supplementary material for: Prevalence, Contamination Level, and Associated Factors of Methicillin-Resistant Staphylococcus aureus in Raw Cow Milk at Selected Districts of Gamo Zone, Southern Ethiopia
Source: Vet Med Int. 2023 Apr 15;2023:6238754. doi: 10.1155/2023/6238754 (PMC10122580; doi:10.1155/2023/6238754)
Supplement: Supplementary Materials — Table data 1. The entire necessary datum are included in the supplementary materials. [file 6238754.f1.pdf]

Table data 1:The entire necessary datum

No	agroecology	TVC count	meanlogTVC T
1	highland	with in average acceptability threshhold/marginal	5.13
2	highland	satisfactory/good for consumption/GMP	4.01
3	highland	with in average acceptability threshhold/marginal	6.92
4	highland	satisfactory/good for consumption/GMP	2.23
5	highland	with in average acceptability threshhold/marginal	6.66
6	highland	with in average acceptability threshhold/marginal	6.02
7	highland	satisfactory/good for consumption/GMP	4.23
8	highland	with in average acceptability threshhold/marginal	5.99
9	highland	satisfactory/good for consumption/GMP	4.11
10	highland	with in average acceptability threshhold/marginal	6.3
11	highland	satisfactory/good for consumption/GMP	3.23
12	highland	satisfactory/good for consumption/GMP	3.08
13	highland	with in average acceptability threshhold/marginal	6.43
14	highland	satisfactory/good for consumption/GMP	2.92
15	highland	with in average acceptability threshhold/marginal	6.94
16	highland	with in average acceptability threshhold/marginal	5.89
17	highland	satisfactory/good for consumption/GMP	4.3
18	highland	satisfactory/good for consumption/GMP	4.86
19	highland	with in average acceptability threshhold/marginal	6.57
20	highland	with in average acceptability threshhold/marginal	6.45
21	highland	satisfactory/good for consumption/GMP	4.12
22	highland	with in average acceptability threshhold/marginal	6.83
23	highland	with in average acceptability threshhold/marginal	5.01
24	highland	unsatisfactory	7
25	highland	satisfactory/good for consumption/GMP	4.6
26	highland	satisfactory/good for consumption/GMP	5
27	highland	satisfactory/good for consumption/GMP	4.9
28	highland	with in average acceptability threshhold/marginal	5.02
29	highland	unsatisfactory	8
30	highland	satisfactory/good for consumption/GMP	4.87
31	highland	unsatisfactory	7.05
32	highland	satisfactory/good for consumption/GMP	4.07
33	highland	with in average acceptability threshhold/marginal	6.1
34	highland	unsatisfactory	7.23
35	highland	unsatisfactory	8.01
36	highland	with in average acceptability threshhold/marginal	6.03
37	highland	with in average acceptability threshhold/marginal	5.78
38	highland	with in average acceptability threshhold/marginal	6.2
39	highland	unsatisfactory	7.12
40	highland	unsatisfactory	7.3
41	highland	with in average acceptability threshhold/marginal	6.34
42	highland	satisfactory/good for consumption/GMP	2.65
43	highland	with in average acceptability threshhold/marginal	5.8
44	highland	satisfactory/good for consumption/GMP	x>8.7
45	highland	with in average acceptability threshhold/marginal	5.31
46	highland	with in average acceptability threshhold/marginal	6.2
47	highland	satisfactory/good for consumption/GMP	4.1

48	highland	with in average acceptability threshhold/marginal	5.76
49	highland	satisfactory/good for consumption/GMP	4.67
50	highland	unsatisfactory	7.02
51	highland	with in average acceptability threshhold/marginal	5.77
52	highland	with in average acceptability threshhold/marginal	5.73
53	highland	with in average acceptability threshhold/marginal	6.02
54	highland	satisfactory/good for consumption/GMP	4.5
55	highland	unsatisfactory	7.2
56	highland	with in average acceptability threshhold/marginal	6.1
57	highland	with in average acceptability threshhold/marginal	6.7
58	highland	with in average acceptability threshhold/marginal	6.23
59	highland	with in average acceptability threshhold/marginal	5.11
60	highland	satisfactory/good for consumption/GMP	3.87
61	lowland	satisfactory/good for consumption/GMP	3.53
62	lowland	with in average acceptability threshhold/marginal	5.88
63	lowland	satisfactory/good for consumption/GMP	4.22
64	lowland	with in average acceptability threshhold/marginal	6.15
65	lowland	satisfactory/good for consumption/GMP	2.12
66	lowland	with in average acceptability threshhold/marginal	6.65
67	lowland	satisfactory/good for consumption/GMP	4.12
68	lowland	with in average acceptability threshhold/marginal	5.74
69	lowland	satisfactory/good for consumption/GMP	2.34
70	lowland	with in average acceptability threshhold/marginal	6.43
71	lowland	satisfactory/good for consumption/GMP	1.09
72	lowland	satisfactory/good for consumption/GMP	3.12
73	lowland	with in average acceptability threshhold/marginal	5.78
74	lowland	satisfactory/good for consumption/GMP	4.01
75	lowland	unsatisfactory	8.52
76	lowland	unsatisfactory	7.45
77	lowland	with in average acceptability threshhold/marginal	6.11
78	lowland	satisfactory/good for consumption/GMP	4.22
79	lowland	with in average acceptability threshhold/marginal	5.81
80	lowland	with in average acceptability threshhold/marginal	6.43
81	lowland	satisfactory/good for consumption/GMP	2.02
82	lowland	with in average acceptability threshhold/marginal	5.32
83	lowland	with in average acceptability threshhold/marginal	6.09
84	lowland	satisfactory/good for consumption/GMP	4.23
85	lowland	with in average acceptability threshhold/marginal	6.98
86	lowland	with in average acceptability threshhold/marginal	5.87
87	lowland	unsatisfactory	8.23
88	lowland	satisfactory/good for consumption/GMP	1.23
89	lowland	satisfactory/good for consumption/GMP	6.7<x?8.7
90	lowland	unsatisfactory	7.98
91	lowland	satisfactory/good for consumption/GMP	4.11
92	lowland	with in average acceptability threshhold/marginal	5.85
93	lowland	with in average acceptability threshhold/marginal	5.92
94	lowland	satisfactory/good for consumption/GMP	3.31
95	lowland	satisfactory/good for consumption/GMP	3.12
96	lowland	unsatisfactory	7.99
97	lowland	with in average acceptability threshhold/marginal	6.03

98	lowland	with in average acceptability threshhold/marginal	5.11
99	lowland	satisfactory/good for consumption/GMP	4.99
100	lowland	with in average acceptability threshhold/marginal	6.23
101	lowland	unsatisfactory	8.09
102	lowland	with in average acceptability threshhold/marginal	6.09
103	lowland	unsatisfactory	7.14
104	lowland	with in average acceptability threshhold/marginal	6.23
105	lowland	with in average acceptability threshhold/marginal	6.22
106	lowland	satisfactory/good for consumption/GMP	4.99
107	lowland	satisfactory/good for consumption/GMP	4.44
108	lowland	with in average acceptability threshhold/marginal	5.76
109	lowland	unsatisfactory	7
110	lowland	satisfactory/good for consumption/GMP	3.71
111	lowland	satisfactory/good for consumption/GMP	2.03
112	lowland	with in average acceptability threshhold/marginal	5.93
113	lowland	satisfactory/good for consumption/GMP	3.23
114	lowland	satisfactory/good for consumption/GMP	3.45
115	lowland	satisfactory/good for consumption/GMP	4.88
116	lowland	with in average acceptability threshhold/marginal	5.77
117	lowland	satisfactory/good for consumption/GMP	5
118	lowland	with in average acceptability threshhold/marginal	5.01
119	lowland	with in average acceptability threshhold/marginal	6.2
120	lowland	unsatisfactory	7.66
121	lowland	satisfactory/good for consumption/GMP	2.1
122	lowland	with in average acceptability threshhold/marginal	6.14
123	lowland	satisfactory/good for consumption/GMP	3.03
124	lowland	unsatisfactory	8
125	lowland	satisfactory/good for consumption/GMP	6.7<x?8.7
126	lowland	with in average acceptability threshhold/marginal	6.11
127	lowland	with in average acceptability threshhold/marginal	6.65
128	lowland	satisfactory/good for consumption/GMP	2.09
129	lowland	satisfactory/good for consumption/GMP	2.26
130	lowland	with in average acceptability threshhold/marginal	6.33
131	lowland	unsatisfactory	7.21
132	lowland	with in average acceptability threshhold/marginal	6.97
133	lowland	with in average acceptability threshhold/marginal	6.32
134	lowland	with in average acceptability threshhold/marginal	5.93
135	lowland	unsatisfactory	7.09
136	lowland	unsatisfactory	7.08
137	lowland	with in average acceptability threshhold/marginal	5.75
138	lowland	satisfactory/good for consumption/GMP	2.99
139	lowland	satisfactory/good for consumption/GMP	3.32
140	lowland	satisfactory/good for consumption/GMP	3.45

Supplementary material

SA count	meanlogTSC	presence of S aureus
potentially harmful	4.74	present
satisfactory/good for consumption/GMP	x?2	absent
unsatisfactory	3.71	present
satisfactory/good for consumption/GMP	x?2	absent
with in average acceptability threshhold/marginal	2.34	present
satisfactory/good for consumption/GMP	x?2	absent
satisfactory/good for consumption/GMP	0.54	present
potentially harmful	4.67	present
with in average acceptability threshhold/marginal	2.56	present
satisfactory/good for consumption/GMP	x?2	absent
with in average acceptability threshhold/marginal	2.45	present
satisfactory/good for consumption/GMP	x?2	absent
unsatisfactory	3.78	present
satisfactory/good for consumption/GMP	x?2	absent
with in average acceptability threshhold/marginal	2.43	present
satisfactory/good for consumption/GMP	x?2	absent
with in average acceptability threshhold/marginal	2.98	present
satisfactory/good for consumption/GMP	x?2	absent
unsatisfactory	3.99	present
satisfactory/good for consumption/GMP	x?2	absent
satisfactory/good for consumption/GMP	x?2	absent
satisfactory/good for consumption/GMP	x?2	absent
with in average acceptability threshhold/marginal	3.7<x<4.7	present
unsatisfactory	4	present
satisfactory/good for consumption/GMP	x?2	absent
satisfactory/good for consumption/GMP	x?2	absent
satisfactory/good for consumption/GMP	x?2	absent
unsatisfactory	3.31	present
satisfactory/good for consumption/GMP	x?2	absent
with in average acceptability threshhold/marginal	2.09	present
with in average acceptability threshhold/marginal	2.87	present
satisfactory/good for consumption/GMP	x?2	absent
with in average acceptability threshhold/marginal	2.1	present
satisfactory/good for consumption/GMP	x?2	absent
with in average acceptability threshhold/marginal	2.54	present
satisfactory/good for consumption/GMP	x?2	absent
with in average acceptability threshhold/marginal	2.88	present
with in average acceptability threshhold/marginal	2.86	present
unsatisfactory	x?2	absent
satisfactory/good for consumption/GMP	x?2	absent
satisfactory/good for consumption/GMP	x?2	absent
unsatisfactory	3.77	present
unsatisfactory	3.71	present
satisfactory/good for consumption/GMP	x?2	absent
unsatisfactory	3.99	present
with in average acceptability threshhold/marginal	2.19	present
with in average acceptability threshhold/marginal	2.88	present

satisfactory/good for consumption/GMP	x?2	absent
satisfactory/good for consumption/GMP	x?2	absent
with in average acceptability threshhold/marginal	2.18	present
satisfactory/good for consumption/GMP	x?2	absent
potentially harmful	4.77	present
unsatisfactory	3.12	present
potentially harmful	4.33	present
satisfactory/good for consumption/GMP	x?2	absent
satisfactory/good for consumption/GMP	x?2	absent
satisfactory/good for consumption/GMP	x?2	absent
potentially harmful	4.01	present
satisfactory/good for consumption/GMP	x?2	absent
satisfactory/good for consumption/GMP	x?2	absent
satisfactory/good for consumption/GMP	x?2	absent
potentially harmful	4.06	present
satisfactory/good for consumption/GMP	x?2	absent
potentially harmful	4.55	present
satisfactory/good for consumption/GMP	x?2	absent
satisfactory/good for consumption/GMP	x?2	absent
satisfactory/good for consumption/GMP	x?2	absent
potentially harmful	4.54	present
satisfactory/good for consumption/GMP	x?2	absent
satisfactory/good for consumption/GMP	x?2	absent
satisfactory/good for consumption/GMP	x?2	absent
satisfactory/good for consumption/GMP	x?2	absent
with in average acceptability threshhold/marginal	x?2	absent
satisfactory/good for consumption/GMP	x?2	absent
with in average acceptability threshhold/marginal	2.76	present
satisfactory/good for consumption/GMP	x?2	absent
unsatisfactory	3.76	present
satisfactory/good for consumption/GMP	x?2	absent
satisfactory/good for consumption/GMP	x?2	absent
with in average acceptability threshhold/marginal	2.18	present
satisfactory/good for consumption/GMP	x?2	absent
satisfactory/good for consumption/GMP	x?2	absent
satisfactory/good for consumption/GMP	x?2	absent
satisfactory/good for consumption/GMP	x?2	absent
potentially harmful	4.35	present
satisfactory/good for consumption/GMP	x?2	absent
satisfactory/good for consumption/GMP	x?2	absent
potentially harmful	4.66	present
satisfactory/good for consumption/GMP	x?2	absent
unsatisfactory	3.34	present
satisfactory/good for consumption/GMP	x?2	absent
satisfactory/good for consumption/GMP	x?2	absent
potentially harmful	4.99	present
satisfactory/good for consumption/GMP	x?2	absent
satisfactory/good for consumption/GMP	1.93	present
satisfactory/good for consumption/GMP	x?2	absent
satisfactory/good for consumption/GMP	x?2	absent

potentially harmful	4.29	present
satisfactory/good for consumption/GMP	x?2	absent
satisfactory/good for consumption/GMP	x?2	absent
potentially harmful	4.11	present
satisfactory/good for consumption/GMP	x?2	absent
satisfactory/good for consumption/GMP	x?2	absent
satisfactory/good for consumption/GMP	x?2	absent
unsatisfactory	3.91	present
satisfactory/good for consumption/GMP	x?2	absent
satisfactory/good for consumption/GMP	x?2	absent
satisfactory/good for consumption/GMP	1.76	present
satisfactory/good for consumption/GMP	x?2	absent
satisfactory/good for consumption/GMP	x?2	absent
satisfactory/good for consumption/GMP	x?2	absent
potentially harmful	4.01	present
satisfactory/good for consumption/GMP	x?2	absent
satisfactory/good for consumption/GMP	x?2	absent
with in average acceptability threshhold/marginal	2.43	present
satisfactory/good for consumption/GMP	x?2	absent
with in average acceptability threshhold/marginal	2.59	present
satisfactory/good for consumption/GMP	x?2	absent
satisfactory/good for consumption/GMP	x?2	absent
potentially harmful	4.21	present
satisfactory/good for consumption/GMP	x?2	absent
with in average acceptability threshhold/marginal	2.45	present
satisfactory/good for consumption/GMP	x?2	absent
satisfactory/good for consumption/GMP	x?2	absent
satisfactory/good for consumption/GMP	x?2	absent
with in average acceptability threshhold/marginal	2.76	present
satisfactory/good for consumption/GMP	x?2	absent
with in average acceptability threshhold/marginal	2.49	present
satisfactory/good for consumption/GMP	x?2	absent
satisfactory/good for consumption/GMP	x?2	absent
with in average acceptability threshhold/marginal	2.99	present
satisfactory/good for consumption/GMP	x?2	absent
potentially harmful	4.38	present
with in average acceptability threshhold/marginal	2.09	present
with in average acceptability threshhold/marginal	2.43	present
with in average acceptability threshhold/marginal	2.64	present
with in average acceptability threshhold/marginal	2.76	present
satisfactory/good for consumption/GMP	x?2	absent
satisfactory/good for consumption/GMP	x?2	absent
unsatisfactory	3.87	present

S.aurous response to PEN	S.aureus response to CXT	S.aureus response to Gen
resistance	susceptible	resistance
no pathogen	no pathogen	no pathogen
no pathogen	no pathogen	no pathogen
susceptible	susceptible	susceptible
resistance	resistance	resistance
no pathogen	no pathogen	no pathogen
resistance	resistance	resistance
no pathogen	no pathogen	no pathogen
resistance	susceptible	resistance
no pathogen	no pathogen	no pathogen
resistance	resistance	susceptible
no pathogen	no pathogen	no pathogen
resistance	resistance	resistance
no pathogen	no pathogen	no pathogen
resistance	resistance	resistance
no pathogen	no pathogen	no pathogen
resistance	resistance	susceptible
no pathogen	no pathogen	no pathogen
resistance	resistance	resistance
no pathogen	no pathogen	no pathogen
susceptible	susceptible	susceptible
no pathogen	no pathogen	no pathogen
resistance	resistance	susceptible
susceptible	resistance	resistance
no pathogen	no pathogen	no pathogen
resistance	susceptible	susceptible
no pathogen	no pathogen	no pathogen
susceptible	resistance	susceptible
no pathogen	no pathogen	no pathogen
resistance	resistance	resistance
resistance	resistance	susceptible
no pathogen	no pathogen	no pathogen
resistance	resistance	resistance
no pathogen	no pathogen	no pathogen
resistance	susceptible	resistance
no pathogen	no pathogen	no pathogen
resistance	resistance	resistance
resistance	resistance	susceptible
no pathogen	no pathogen	no pathogen
no pathogen	no pathogen	no pathogen
no pathogen	no pathogen	no pathogen
resistance	resistance	resistance
susceptible	susceptible	susceptible
no pathogen	no pathogen	no pathogen
resistance	resistance	resistance
resistance	resistance	susceptible
resistance	resistance	resistance

no pathogen	no pathogen	no pathogen
no pathogen	no pathogen	no pathogen
resistance	resistance	susceptible
no pathogen	no pathogen	no pathogen
resistance	resistance	resistance
resistance	resistance	susceptible
resistance	resistance	resistance
no pathogen	no pathogen	no pathogen
no pathogen	no pathogen	no pathogen
no pathogen	no pathogen	no pathogen
resistance	resistance	resistance
susceptible	susceptible	susceptible
no pathogen	no pathogen	no pathogen
no pathogen	no pathogen	no pathogen
resistance	resistance	susceptible
no pathogen	no pathogen	no pathogen
resistance	resistance	resistance
no pathogen	no pathogen	no pathogen
no pathogen	no pathogen	no pathogen
no pathogen	no pathogen	no pathogen
resistance	resistance	resistance
no pathogen	no pathogen	no pathogen
no pathogen	no pathogen	no pathogen
susceptible	susceptible	susceptible
no pathogen	no pathogen	no pathogen
no pathogen	no pathogen	no pathogen
no pathogen	no pathogen	no pathogen
resistance	resistance	susceptible
no pathogen	no pathogen	no pathogen
resistance	resistance	susceptible
no pathogen	no pathogen	no pathogen
no pathogen	no pathogen	no pathogen
resistance	resistance	susceptible
no pathogen	no pathogen	no pathogen
susceptible	susceptible	susceptible
no pathogen	no pathogen	no pathogen
no pathogen	no pathogen	no pathogen
resistance	resistance	resistance
no pathogen	no pathogen	no pathogen
no pathogen	no pathogen	no pathogen
no pathogen	no pathogen	no pathogen
no pathogen	no pathogen	no pathogen
resistance	resistance	susceptible
no pathogen	no pathogen	no pathogen
no pathogen	no pathogen	no pathogen
resistance	resistance	resistance
no pathogen	no pathogen	no pathogen
resistance	susceptible	resistance
no pathogen	no pathogen	no pathogen
no pathogen	no pathogen	no pathogen

resistance	resistance	susceptible
no pathogen	no pathogen	no pathogen
no pathogen	no pathogen	no pathogen
resistance	resistance	resistance
no pathogen	no pathogen	no pathogen
no pathogen	no pathogen	no pathogen
susceptible	susceptible	resistance
resistance	resistance	susceptible
no pathogen	no pathogen	no pathogen
no pathogen	no pathogen	no pathogen
resistance	resistance	resistance
no pathogen	no pathogen	no pathogen
no pathogen	no pathogen	no pathogen
no pathogen	no pathogen	no pathogen
no pathogen	no pathogen	no pathogen
no pathogen	no pathogen	no pathogen
no pathogen	no pathogen	no pathogen
resistance	resistance	susceptible
no pathogen	no pathogen	no pathogen
resistance	resistance	resistance
no pathogen	no pathogen	no pathogen
no pathogen	no pathogen	no pathogen
resistance	resistance	susceptible
no pathogen	no pathogen	no pathogen
no pathogen	no pathogen	no pathogen
no pathogen	no pathogen	no pathogen
no pathogen	no pathogen	no pathogen
no pathogen	no pathogen	no pathogen
resistance	resistance	susceptible
no pathogen	no pathogen	no pathogen
resistance	susceptible	resistance
no pathogen	no pathogen	no pathogen
no pathogen	no pathogen	no pathogen
resistance	resistance	resistance
no pathogen	no pathogen	no pathogen
no pathogen	no pathogen	no pathogen
resistance	resistance	susceptible
resistance	susceptible	resistance
no pathogen	no pathogen	no pathogen
resistance	resistance	susceptible
no pathogen	no pathogen	no pathogen
no pathogen	no pathogen	no pathogen
resistance	resistance	susceptible

S.aureus response toEry	S.aureus response to TET	S.aureus response toCIP
intermediate	no pathogen	susceptible
no pathogen	susceptible	no pathogen
no pathogen	resistance	no pathogen
susceptible	no pathogen	susceptible
susceptible	intermediate	susceptible
no pathogen	no pathogen	no pathogen
intermediate	resistance	susceptible
no pathogen	no pathogen	no pathogen
resistance	susceptible	susceptible
no pathogen	no pathogen	no pathogen
susceptible	intermediate	susceptible
no pathogen	no pathogen	no pathogen
susceptible	resistance	susceptible
no pathogen	no pathogen	no pathogen
resistance	susceptible	susceptible
no pathogen	no pathogen	no pathogen
susceptible	resistance	susceptible
no pathogen	no pathogen	no pathogen
susceptible	intermediate	susceptible
no pathogen	no pathogen	no pathogen
susceptible	resistance	susceptible
no pathogen	susceptible	no pathogen
susceptible	no pathogen	susceptible
susceptible	susceptible	susceptible
no pathogen	no pathogen	no pathogen
susceptible	susceptible	susceptible
no pathogen	no pathogen	no pathogen
resistance	resistance	susceptible
no pathogen	susceptible	no pathogen
intermediate	no pathogen	susceptible
susceptible	resistance	susceptible
no pathogen	no pathogen	no pathogen
resistance	resistance	susceptible
no pathogen	no pathogen	no pathogen
susceptible	susceptible	susceptible
no pathogen	resistance	no pathogen
resistance	no pathogen	susceptible
susceptible	no pathogen	resistance
no pathogen	no pathogen	no pathogen
no pathogen	intermediate	no pathogen
no pathogen	susceptible	no pathogen
intermediate	no pathogen	susceptible
resistance	intermediate	susceptible
no pathogen	intermediate	no pathogen
intermediate	resistance	susceptible
resistance	no pathogen	susceptible
resistance	no pathogen	susceptible

no pathogen	resistance	no pathogen
no pathogen	no pathogen	no pathogen
resistance	susceptible	susceptible
no pathogen	resistance	no pathogen
susceptible	intermediate	susceptible
resistance	no pathogen	susceptible
intermediate	no pathogen	resistance
no pathogen	no pathogen	no pathogen
no pathogen	resistance	no pathogen
no pathogen	susceptible	no pathogen
susceptible	no pathogen	susceptible
susceptible	no pathogen	susceptible
no pathogen	susceptible	no pathogen
no pathogen	no pathogen	no pathogen
susceptible	intermediate	susceptible
no pathogen	no pathogen	no pathogen
resistance	no pathogen	susceptible
no pathogen	no pathogen	no pathogen
no pathogen	susceptible	no pathogen
no pathogen	no pathogen	no pathogen
susceptible	no pathogen	susceptible
no pathogen	susceptible	no pathogen
no pathogen	no pathogen	no pathogen
resistance	no pathogen	susceptible
no pathogen	no pathogen	no pathogen
no pathogen	resistance	no pathogen
no pathogen	no pathogen	no pathogen
resistance	susceptible	susceptible
no pathogen	no pathogen	no pathogen
susceptible	no pathogen	susceptible
no pathogen	resistance	no pathogen
no pathogen	no pathogen	no pathogen
susceptible	intermediate	susceptible
no pathogen	no pathogen	no pathogen
susceptible	no pathogen	susceptible
no pathogen	susceptible	no pathogen
no pathogen	no pathogen	no pathogen
intermediate	no pathogen	susceptible
no pathogen	no pathogen	no pathogen
no pathogen	no pathogen	no pathogen
no pathogen	susceptible	no pathogen
no pathogen	no pathogen	no pathogen
resistance	no pathogen	susceptible
no pathogen	intermediate	no pathogen
no pathogen	no pathogen	no pathogen
susceptible	intermediate	susceptible
no pathogen	no pathogen	no pathogen
intermediate	no pathogen	susceptible
no pathogen	susceptible	no pathogen
no pathogen	no pathogen	no pathogen

susceptible	no pathogen	susceptible
no pathogen	intermediate	no pathogen
no pathogen	no pathogen	no pathogen
susceptible	no pathogen	susceptible
no pathogen	susceptible	no pathogen
no pathogen	susceptible	no pathogen
susceptible	no pathogen	susceptible
susceptible	no pathogen	susceptible
no pathogen	susceptible	no pathogen
no pathogen	no pathogen	no pathogen
resistance	no pathogen	susceptible
no pathogen	no pathogen	no pathogen
no pathogen	no pathogen	no pathogen
no pathogen	no pathogen	no pathogen
no pathogen	no pathogen	no pathogen
no pathogen	susceptible	no pathogen
no pathogen	no pathogen	no pathogen
resistance	resistance	susceptible
no pathogen	no pathogen	no pathogen
resistance	no pathogen	susceptible
no pathogen	susceptible	no pathogen
no pathogen	no pathogen	no pathogen
resistance	no pathogen	susceptible
no pathogen	no pathogen	no pathogen
no pathogen	no pathogen	no pathogen
no pathogen	no pathogen	no pathogen
no pathogen	intermediate	no pathogen
no pathogen	no pathogen	no pathogen
resistance	susceptible	susceptible
no pathogen	no pathogen	no pathogen
susceptible	no pathogen	susceptible
no pathogen	susceptible	no pathogen
no pathogen	no pathogen	no pathogen
susceptible	no pathogen	susceptible
no pathogen	susceptible	no pathogen
no pathogen	resistance	no pathogen
resistance	no pathogen	susceptible
susceptible	resistance	susceptible
no pathogen	no pathogen	no pathogen
resistance	no pathogen	susceptible
no pathogen	susceptible	no pathogen
no pathogen		no pathogen
resistance		susceptible

S.aureus response to Cot	S.aureus response to Cll	S.aureus response to CHL
susceptible	susceptible	susceptible
no pathogen	no pathogen	no pathogen
no pathogen	no pathogen	no pathogen
susceptible	susceptible	susceptible
susceptible	susceptible	susceptible
no pathogen	no pathogen	no pathogen
resistance	susceptible	susceptible
no pathogen	no pathogen	no pathogen
susceptible	susceptible	susceptible
no pathogen	no pathogen	no pathogen
susceptible	susceptible	susceptible
no pathogen	no pathogen	no pathogen
susceptible	susceptible	susceptible
no pathogen	no pathogen	no pathogen
susceptible	susceptible	susceptible
no pathogen	no pathogen	no pathogen
susceptible	susceptible	susceptible
no pathogen	no pathogen	no pathogen
susceptible	susceptible	susceptible
no pathogen	no pathogen	no pathogen
susceptible	susceptible	susceptible
no pathogen	no pathogen	no pathogen
susceptible	susceptible	susceptible
susceptible	susceptible	susceptible
no pathogen	no pathogen	no pathogen
susceptible	susceptible	susceptible
no pathogen	no pathogen	no pathogen
susceptible	susceptible	susceptible
no pathogen	no pathogen	no pathogen
susceptible	susceptible	resistance
susceptible	susceptible	susceptible
no pathogen	no pathogen	no pathogen
susceptible	susceptible	susceptible
no pathogen	no pathogen	no pathogen
susceptible	susceptible	resistance
no pathogen	no pathogen	no pathogen
susceptible	susceptible	susceptible
susceptible	susceptible	intermediate
no pathogen	no pathogen	no pathogen
no pathogen	no pathogen	no pathogen
no pathogen	no pathogen	no pathogen
susceptible	susceptible	susceptible
susceptible	susceptible	susceptible
no pathogen	no pathogen	no pathogen
resistance	susceptible	resistance
susceptible	susceptible	susceptible
susceptible	susceptible	susceptible

no pathogen	no pathogen	no pathogen
no pathogen	no pathogen	no pathogen
susceptible	susceptible	susceptible
no pathogen	no pathogen	no pathogen
susceptible	susceptible	susceptible
susceptible	susceptible	susceptible
intermediate	susceptible	resistance
no pathogen	no pathogen	no pathogen
no pathogen	no pathogen	no pathogen
no pathogen	no pathogen	no pathogen
susceptible	susceptible	susceptible
susceptible	susceptible	susceptible
no pathogen	no pathogen	no pathogen
no pathogen	no pathogen	no pathogen
susceptible	susceptible	susceptible
no pathogen	no pathogen	no pathogen
susceptible	susceptible	susceptible
no pathogen	no pathogen	no pathogen
no pathogen	no pathogen	no pathogen
no pathogen	no pathogen	no pathogen
susceptible	susceptible	susceptible
no pathogen	no pathogen	no pathogen
no pathogen	no pathogen	no pathogen
susceptible	susceptible	susceptible
no pathogen	no pathogen	no pathogen
no pathogen	no pathogen	no pathogen
no pathogen	no pathogen	no pathogen
susceptible	resistance	susceptible
no pathogen	no pathogen	no pathogen
susceptible	susceptible	susceptible
no pathogen	no pathogen	no pathogen
no pathogen	no pathogen	no pathogen
susceptible	susceptible	susceptible
no pathogen	no pathogen	no pathogen
susceptible	susceptible	susceptible
no pathogen	no pathogen	no pathogen
no pathogen	no pathogen	no pathogen
susceptible	susceptible	susceptible
no pathogen	no pathogen	no pathogen
no pathogen	no pathogen	no pathogen
no pathogen	no pathogen	no pathogen
no pathogen	no pathogen	no pathogen
susceptible	susceptible	susceptible
no pathogen	no pathogen	no pathogen
no pathogen	no pathogen	no pathogen
susceptible	susceptible	susceptible
no pathogen	no pathogen	no pathogen
susceptible	susceptible	resistance
no pathogen	no pathogen	no pathogen
no pathogen	no pathogen	no pathogen

susceptible	susceptible	susceptible
no pathogen	no pathogen	no pathogen
no pathogen	no pathogen	no pathogen
susceptible	susceptible	susceptible
no pathogen	no pathogen	no pathogen
no pathogen	no pathogen	no pathogen
susceptible	susceptible	susceptible
susceptible	susceptible	susceptible
no pathogen	no pathogen	no pathogen
no pathogen	no pathogen	no pathogen
susceptible	resistance	susceptible
no pathogen	no pathogen	no pathogen
no pathogen	no pathogen	no pathogen
no pathogen	no pathogen	no pathogen
no pathogen	no pathogen	no pathogen
no pathogen	no pathogen	no pathogen
no pathogen	no pathogen	no pathogen
susceptible	susceptible	susceptible
no pathogen	no pathogen	no pathogen
susceptible	susceptible	susceptible
no pathogen	no pathogen	no pathogen
no pathogen	no pathogen	no pathogen
susceptible	susceptible	susceptible
no pathogen	no pathogen	no pathogen
no pathogen	no pathogen	no pathogen
no pathogen	no pathogen	no pathogen
no pathogen	no pathogen	no pathogen
no pathogen	no pathogen	no pathogen
susceptible	susceptible	susceptible
no pathogen	no pathogen	no pathogen
susceptible	susceptible	susceptible
no pathogen	no pathogen	no pathogen
no pathogen	no pathogen	no pathogen
susceptible	susceptible	susceptible
no pathogen	no pathogen	no pathogen
no pathogen	no pathogen	no pathogen
susceptible	susceptible	susceptible
susceptible	resistance	susceptible
no pathogen	no pathogen	no pathogen
susceptible	susceptible	susceptible
no pathogen	no pathogen	no pathogen
no pathogen	no pathogen	no pathogen
susceptible	susceptible	susceptible

sex of the respondant	educational status of workers on milk	training of workers
female	01-Aug	no
female	college and above	no
female	ilietrate	no
female	09-Dec	no
female	01-Aug	no
female	01-Aug	no
female	01-Aug	no
female	ilietrate	no
male	01-Aug	no
male	college and above	no
male	01-Aug	no
male	01-Aug	no
female	01-Aug	no
female	09-Dec	no
female	01-Aug	no
female	college and above	no
male	ilietrate	no
male	01-Aug	no
male	01-Aug	no
male	01-Aug	no
female	01-Aug	no
female	09-Dec	no
female	01-Aug	no
female	01-Aug	no
female	09-Dec	no
female	01-Aug	no
female	college and above	no
female	01-Aug	no
male	01-Aug	no
male	ilietrate	no
male	01-Aug	no
male	09-Dec	no
female	01-Aug	no
female	01-Aug	no
female	01-Aug	no
female	01-Aug	no
female	ilietrate	no
female	01-Aug	no
female	01-Aug	no
female	college and above	no
female	09-Dec	no
female	01-Aug	no
female	01-Aug	no
female	college and above	no
female	01-Aug	no
female	ilietrate	no
female	01-Aug	no

female	01-Aug	no
male	09-Dec	no
male	01-Aug	no
male	01-Aug	no
male	01-Aug	no
male	01-Aug	no
male	ilietrate	no
male	college and above	no
male	09-Dec	no
female	01-Aug	no
female	ilietrate	no
female	college and above	no
female	01-Aug	no
male	09-Dec	no
male	ilietrate	no
male	01-Aug	no
male	01-Aug	no
female	09-Dec	no
female	01-Aug	no
female	01-Aug	no
female	ilietrate	no
female	college and above	no
female	01-Aug	no
female	09-Dec	no
female	01-Aug	no
male	01-Aug	yes
male	01-Aug	no
male	01-Aug	no
male	college and above	no
male	01-Aug	no
male	09-Dec	no
male	01-Aug	yes
male	ilietrate	no
female	01-Aug	no
female	09-Dec	no
female	09-Dec	no
female	01-Aug	no
female	01-Aug	no
female	01-Aug	no
female	ilietrate	no
female	01-Aug	no
male	college and above	yes
male	01-Aug	no
male	01-Aug	no
male	09-Dec	no
female	01-Aug	no
female	01-Aug	no
female	college and above	no
female	09-Dec	yes
female	01-Aug	no

female	01-Aug	no
female	01-Aug	no
female	09-Dec	no
female	ilietrate	no
female	college and above	no
female	01-Aug	yes
female	01-Aug	no
female	09-Dec	no
female	01-Aug	no
female	01-Aug	no
female	ilietrate	no
male	09-Dec	yes
male	01-Aug	no
male	01-Aug	no
male	01-Aug	no
female	01-Aug	no
female	college and above	yes
female	ilietrate	no
female	college and above	no
female	ilietrate	no
female	09-Dec	no
female	college and above	yes
female	01-Aug	no
male	college and above	no
male	01-Aug	no
male	ilietrate	no
male	college and above	yes
female	09-Dec	no
female	01-Aug	no
female	09-Dec	yes
female	ilietrate	no
female	01-Aug	no
female	college and above	no
female	01-Aug	no
female	09-Dec	yes
female	01-Aug	no
female	ilietrate	no
female	01-Aug	no
female	09-Dec	no
male	01-Aug	no
male	college and above	yes
male	01-Aug	no
male	01-Aug	no

hair covered with hai rcover	wearing of clean over coat/apron	health check up
no	no	no
no	no	no
no	no	no
yes	no	no
no	no	no
no	no	no
no	no	no
no	no	no
no	no	no
no	no	no
no	no	no
no	no	no
yes	no	no
no	no	no
no	no	no
no	no	no
no	yes	no
no	yes	no
no	yes	no
yes	yes	no
no	no	no
no	no	no
no	no	no
no	no	no
yes	no	no
no	no	no
no	no	no
no	no	no
no	no	no
no	no	no
no	no	no
no	no	no
no	no	no
no	no	no
no	no	no
no	no	no
no	yes	no
no	yes	no
no	yes	no
no	yes	no
no	no	no
no	no	no
no	no	no
no	no	no
no	no	no
no	no	no
no	no	no

no	no	no
no	no	no
no	no	no
no	no	no
no	no	no
no	no	no
no	no	no
no	no	no
no	no	no
no	yes	no
no	yes	no
no	yes	no
no	yes	no
no	no	no
no	no	no
no	no	no
no	no	no
no	no	no
no	no	no
no	no	no
no	no	no
no	no	no
no	no	no
no	no	no
no	no	no
no	no	no
no	no	no
no	no	no
no	no	no
no	no	no
no	no	no
no	no	no
no	no	no
yes	no	no
yes	no	no
yes	no	no
yes	no	no
no	no	no
no	no	no
no	yes	no
no	yes	no
no	yes	no
no	yes	no
no	no	no
no	no	no
no	no	no
no	no	no
no	yes	no
no	yes	no
no	yes	no

no	yes	no
no	no	no
no	no	no
no	no	no
no	no	no
no	no	no
no	no	no
no	no	no
no	no	no
no	no	no
no	no	no
yes	no	no
yes	no	no
yes	yes	no
yes	yes	no
no	yes	no
no	yes	no
no	no	no
no	no	no
no	no	no
no	no	no
no	no	no
no	no	no
yes	no	yes
yes	no	yes
yes	no	yes
yes	no	yes
no	no	no
no	no	no
no	no	no
no	no	no
no	no	no
no	no	no
no	yes	no
no	yes	no
yes	yes	yes
yes	yes	yes
yes	no	yes
yes	no	yes
no	no	no
no	no	no
no	no	no
no	no	no

nose picking habit of the workers during working	washing hands before and after
no	water
yes	water
yes	water
yes	water
no	water
yes	detergent with water
yes	water
no	water
no	water
yes	water
no	water
yes	detergent with water
no	water
yes	water
no	water
yes	water
yes	water
yes	water
yes	water
no	water
yes	detergent with water
yes	water
no	detergent with water
yes	water
no	detergent with water
no	detergent with water
yes	detergent with water
yes	water
yes	detergent with water
no	water
no	detergent with water
yes	detergent with water
no	water
yes	water
no	water
yes	detergent with water
no	detergent with water
no	detergent with water
yes	water
yes	detergent with water
yes	detergent with water
yes	water
no	water
yes	detergent with water
no	detergent with water
no	water
no	water

yes	detergent with water
yes	water
no	water
yes	detergent with water
no	water
yes	water
yes	detergent with water
yes	detergent with water
yes	water
yes	detergent with water
no	water
yes	detergent with water
yes	detergent with water
yes	water
no	water
yes	detergent with water
no	water
yes	detergent with water
yes	water
yes	detergent with water
no	water
yes	detergent with water
yes	water
no	detergent with water
no	detergent with water
yes	water
yes	water
no	detergent with water
yes	detergent with water
yes	detergent with water
no	detergent with water
yes	detergent with water
no	water
yes	detergent with water
no	water
no	detergent with water
yes	water
no	water
yes	water
no	water
no	water
yes	water
no	water
yes	detergent with water
yes	water
no	water
yes	detergent with water
no	water
yes	detergent with water
no	water

no	water
yes	detergent with water
yes	detergent with water
no	water
yes	detergent with water
no	detergent with water
no	water
yes	water
yes	detergent with water
no	detergent with water
no	water
yes	detergent with water
yes	water
yes	detergent with water
no	water
yes	detergent with water
yes	detergent with water
no	water
yes	detergent with water
no	water
yes	detergent with water
yes	detergent with water
yes	water
no	water
yes	water
no	detergent with water
yes	detergent with water
yes	detergent with water
no	water
yes	detergent with water
no	water
no	detergent with water
no	detergent with water
yes	water
yes	detergent with water
yes	water
yes	detergent with water
no	water
yes	water
no	water
no	detergent with water
yes	detergent with water
no	detergent with water

cleaning milk container	abnormal milk checking	milk container
detergent with water	no	plastic
detergent with water	yes	plastic
water	no	plastic
detergent with water	yes	aluminium coated vessels
water	no	plastic
detergent with water	yes	plastic
water	yes	plastic
water	no	plastic
water	yes	plastic
detergent with water	yes	plastic
water	no	plastic
detergent with water	yes	plastic
water	no	plastic
detergent with water	yes	plastic
water	no	plastic
detergent with water	yes	plastic
water	no	plastic
detergent with water	yes	aluminium coated vessels
water	no	plastic
detergent with water	yes	plastic
detergent with water	yes	plastic
detergent with water	yes	plastic
water	no	plastic
detergent with water	no	plastic
detergent with water	yes	aluminium coated vessels
water	yes	plastic
detergent with water	yes	aluminium coated vessels
water	yes	aluminium coated vessels
detergent with water	yes	plastic
water	no	plastic
detergent with water	yes	plastic
detergent with water	yes	aluminium coated vessels
water	no	plastic
detergent with water	yes	aluminium coated vessels
water	no	aluminium coated vessels
detergent with water	yes	plastic
water	yes	plastic
detergent with water	yes	plastic
detergent with water	no	plastic
detergent with water	yes	aluminium coated vessels
detergent with water	yes	aluminium coated vessels
water	yes	plastic
water	no	plastic
detergent with water	yes	aluminium coated vessels
water	no	plastic
water	yes	plastic
water	no	plastic

detergent with water	yes	plastic
detergent with water	yes	plastic
water	yes	aluminium coated vessels
detergent with water	yes	plastic
water	yes	aluminium coated vessels
water	yes	plastic
water	no	plastic
detergent with water	yes	aluminium coated vessels
detergent with water	yes	aluminium coated vessels
detergent with water	yes	aluminium coated vessels
water	no	plastic
detergent with water	yes	plastic
detergent with water	yes	plastic
detergent with water	yes	aluminium coated vessels
water	no	plastic
detergent with water	yes	plastic
water	no	aluminium coated vessels
detergent with water	yes	aluminium coated vessels
detergent with water	no	plastic
detergent with water	yes	plastic
water	no	plastic
detergent with water	yes	aluminium coated vessels
detergent with water	no	plastic
detergent with water	yes	plastic
detergent with water	yes	aluminium coated vessels
detergent with water	no	aluminium coated vessels
water	no	plastic
water	no	aluminium coated vessels
detergent with water	yes	plastic
water	no	plastic
detergent with water	no	aluminium coated vessels
detergent with water	yes	plastic
water	no	plastic
detergent with water	yes	aluminium coated vessels
detergent with water	yes	plastic
water	yes	plastic
detergent with water	yes	aluminium coated vessels
water	no	plastic
detergent with water	yes	plastic
detergent with water	yes	aluminium coated vessels
water	no	plastic
detergent with water	yes	plastic
water	no	plastic
detergent with water	yes	plastic
detergent with water	no	aluminium coated vessels
water	no	plastic
detergent with water	yes	plastic
water	no	aluminium coated vessels
detergent with water	yes	plastic
water	no	plastic

water	no	plastic
water	no	plastic
detergent with water	yes	aluminium coated vessels
water	no	plastic
detergent with water	yes	aluminium coated vessels
detergent with water	yes	plastic
water	yes	plastic
water	yes	plastic
detergent with water	yes	plastic
detergent with water	yes	aluminium coated vessels
water	no	plastic
detergent with water	yes	aluminium coated vessels
water	no	aluminium coated vessels
detergent with water	yes	plastic
water	no	plastic
water	yes	aluminium coated vessels
detergent with water	yes	aluminium coated vessels
water	yes	plastic
detergent with water	yes	aluminium coated vessels
detergent with water	no	plastic
water	yes	aluminium coated vessels
detergent with water	no	plastic
water	yes	plastic
detergent with water	yes	aluminium coated vessels
detergent with water	no	plastic
water	yes	plastic
detergent with water	yes	aluminium coated vessels
detergent with water	yes	plastic
water	no	plastic
detergent with water	yes	aluminium coated vessels
water	no	plastic
detergent with water	yes	plastic
detergent with water	yes	aluminium coated vessels
water	no	plastic
water	yes	plastic
detergent with water	no	plastic
water	no	aluminium coated vessels
detergent with water	yes	plastic
detergent with water	yes	plastic
water	no	plastic
detergent with water	yes	aluminium coated vessels
water	no	aluminium coated vessels
water	no	plastic
